# Treadmilling and dynamic protrusions in fire ant rafts

**DOI:** 10.1098/rsif.2021.0213

**Published:** 2021-06-30

**Authors:** Robert J. Wagner, Kristen Such, Ethan Hobbs, Franck J. Vernerey

**Affiliations:** ^1^ Mechanical Engineering Department, Material Science and Engineering Program, University of Colorado, Boulder, CO 80309 USA; ^2^ Mechanical Engineering Department, University of Colorado, Boulder, CO 80309 USA; ^3^ Computer Science Department, Interdisciplinary Quantitative Biology Program, University of Colorado, Boulder, CO 80309 USA

**Keywords:** cooperative behaviour, emergent, non-equilibrium, dynamic networks, self-propelled

## Abstract

Fire ants (*Solenopsis invicta*) are exemplary for their formation of cohered, buoyant and dynamic structures composed entirely of their own bodies when exposed to flooded environments. Here, we observe tether-like protrusions that emerge from aggregated fire ant rafts when docked to stationary, vertical rods. Ant rafts comprise a floating, structural network of interconnected ants on which a layer of freely active ants walk. We show here that sustained shape evolution is permitted by the competing mechanisms of perpetual raft contraction aided by the transition of bulk structural ants to the free active layer and outward raft expansion owing to the deposition of free ants into the structural network at the edges, culminating in global treadmilling. Furthermore, we see that protrusions emerge as a result of asymmetries in the edge deposition rate of free ants. Employing both experimental characterization and a model for self-propelled particles in strong confinement, we interpret that these asymmetries are likely to occur stochastically owing to wall accumulation effects and directional motion of active ants when strongly confined by the protrusions' relatively narrow boundaries. Together, these effects may realize the cooperative, yet spontaneous formation of protrusions that fire ants sometimes use for functional exploration and to escape flooded environments.

## 1. Introduction

Collective emergent behaviour is a remarkable and omnipresent feature of living systems that often results in functions such as motility of aggregations, self-healing of tissues and morphing of swarms [1–3]. Cooperatively behaving living systems are of interest to a wide variety of researchers ranging from biologists [4] and physicists [5] to engineers [6] and roboticists [7], because they elucidate the local-to-global relationship in complex ecologies or physical systems and may inspire a broad class of functional metamaterials that adapt their mechanical properties or autonomously self-assemble. One category of organisms, favourably studied for their macroscopic size and ease of observation, is insect aggregations [8,9], including those of the red imported fire ant (*Solenopsis invicta*). Fire ants condense into buoyant rafts comprising worker ant bodies when their habitats are flooded. These cohesive swarms are cross-linked by reversible ant-to-ant bonds [10–12] which may dissociate from highly stressed states and re-associate into lower energy configurations without sustaining damage. In the last 10 years, researchers have begun to investigate the mechanical properties of these aggregated swarms, which demonstrate nonlinear viscoelastic responses because of the reversibility of their inter-ant bonds [9,13,14]. However, another remarkable feature of fire ants that contributes to their complex response is activity [15,16].

**Figure 1.**
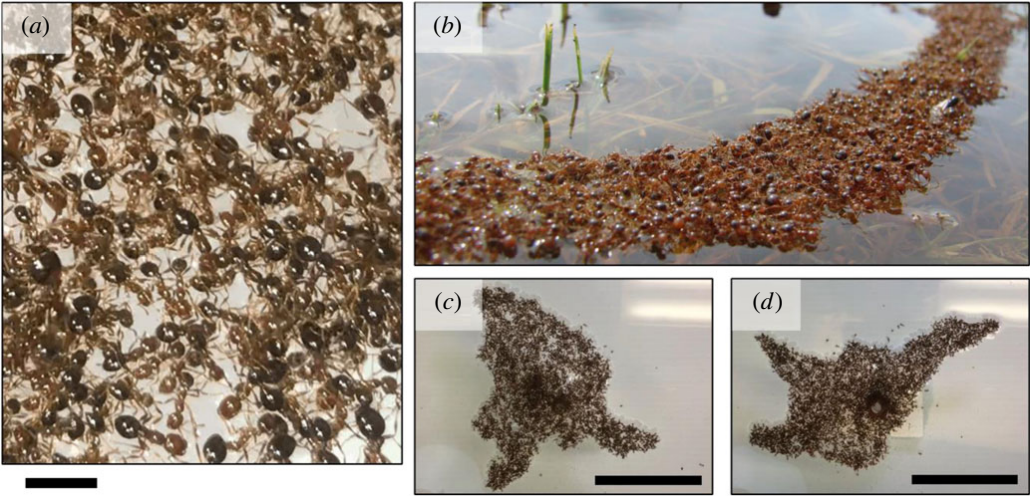
Networked fire ant rafts form dynamic structures: (*a*) the floating, structural network of ant rafts is cross-linked by ant-to-ant bonds. The scale bar represents 1 ℓ or one average ant body length. (*b*) Fire ants in nature form rafts that, under various boundary conditions, create tether-like protrusions and bridges. Photo used with the permission of Alison A. Bockoven, Arizona Western College (alison.bockoven@azwestern.edu). A top view of an experimental raft anchored to an acrylic rod is depicted at the (*c*) start and (*d*) end of an approximately 60 min duration to illustrate the cyclical protrusion growth that occurs over hour time scales. Scale bars represent 20 ℓ*.*

Individual ants convert chemical energy into mechanical work, including both locomotion and—as with many active systems [17,18]—active contraction. This activity endows unperturbed fire ants with the ability to dynamically change their raft shapes [19] and even form complex, three-dimensional (3D) structures such as towers nucleated about substrates [20]. While the behaviour and flow of 3D ant towers has been examined [20,21], questions remain regarding the long-term dynamics of approximately two-dimensional (2D) rafts. Mlot *et al.* [11] reported that, upon being placed into the water as approximately 3D spheroids, ant rafts spread out rapidly. Confirming these observations and those of Adams *et al.* [12], we see that ant rafts consist of a bounded network of interconnected structural ants that float on the water (figure 1*a*). On the top of this network, a dispersed state of ants walk freely (see electronic supplementary material, Movie S1 for clarity). Mlot *et al.* studied the initial raft expansion over short time scales (up to 200 s) experimentally [11] and intermediate time scales (up to 10^3^ s) numerically [19]. They reported that free ants walk on the surface of the structural raft until they encounter the edge, at which point they either bank off said edge, pause or deposit into the structural layer (see electronic supplementary material, Movie S2 for local observations). This deposition of free ants into the structural network drives outward raft expansion [11,19]. However, here, we observe over longer time scales (>10^3^ s) that ant rafts under specific boundary conditions undergo cyclical and sustained dynamic shape changes, including the formation of 2D tether-like instabilities that protrude from the rafts’ edges (figure 1*b*). These protrusions have, to our knowledge, been neither documented nor explained in the existing literature. Edge deposition, alone, cannot explain the initiation, growth and complete reclamation of protrusions observed (figure 1*c*,*d*). Without any cyclic mechanism(s) of turnover or dynamic properties in the structural network itself, one would expect the shape of the raft to become static once the population of free ants is exhausted. That this is not the case implies either the population of free ants is replenished, the structural network morphs or some combination of both.

Indeed, here we report that the structural networks of ant rafts anchored to vertical rods contract in a process that counteracts edge deposition-driven expansion. Furthermore, this contraction occurs simultaneously with the exiting of some ants from the structural layer, which replenishes the population of free ants (see electronic supplementary material, Movie S3 for observations). These competing mechanisms balance rafts into a pseudo-steady state of torus-like treadmilling (electronic supplementary material, Movie S4) that vaguely resembles the phenomena observed in cytoskeletal systems of actin filaments [22]. As in the case of cytoskeletal systems [23], this treadmilling leads to the cyclical turnover of constituents that facilitates sustained shape change, which in ant rafts includes periods of unstable protrusion growth. In the remainder of this work, we detail our experimental and data-processing methods. We then report on the dynamic properties of both free and structural ants, including the rates of transition between these respective states. Finally, we examine the local properties of self-propelled free ants on protrusions to reveal that directed motion occurs in these strongly confined regions. Employing a model for strongly confined self-propelled particles (SPPs), we interpret that both density gradients along the rafts' edges and confinement-induced directed motion of ants on protrusions are likely to contribute to the runaway growth of instabilities.

## 2. Methods

### 2.1. Experimental design

To conduct experiments, we collected 3–10 g of worker ants (or approx. 3000–10 000 ants) and placed them into a container of water, where they enveloped and nucleated to a stationary acrylic rod protruding vertically from the waterline. Both Ø6 mm and Ø16 mm rods were tested with and without a talcum powder coating to prohibit climbing. The degree to which the rods protruded from the water's surface was varied from less than 1 to 15 cm. Treadmilling and instabilities were observed under all boundary conditions over the span of several hours until many of the free ants became inactive and clustered near the rod. In the scope of this work, sampling was performed sufficiently far from the rod so that inactive free ants were not characterized since they did not contribute to raft dynamics. Additionally, sampling was only conducted while enough ants remained active to sustain relatively steady raft dynamics. To mitigate potential temperature effects on activity, air temperature in the room was maintained between 20 and 24°C. The water temperature was monitored and remained between 17.9 and 19.0°C. Cameras were positioned above the rafts to capture footage. Time-lapse footage, captured throughout the entirety of select experiments, was used to characterize structural ants and raft dynamics. Real-time footage, captured every 10 min throughout the duration of the experiments (to ensure representative temporal sampling), was used to characterize free ants. Reference length scales were placed horizontally in the frame, at the water line. Footage was imported into and processed using ImageJ [24–26]. Data post-processing was achieved using Matlab 2019b [27].

### 2.2. Planar density

The planar density of the structural ants constituting the floating layer of the raft (*ρ_r_*) was estimated by counting the number of structural ants residing within regions of a known area. The planar density of free ants that walk on the top of the raft was difficult to measure owing to heterogeneity and clustering. The mean packing fraction of free ants $\left(\;\bar{\;\phi }\right)$ was estimated according to $\;\bar{\;\phi }=(N_{\mathrm{tot}}-A\rho_{r})/A\rho_{r}$, where *N*_tot_ is the total number of ants, *A* is the total raft area and *Aρ_r_* is the number of structural ants given that *ρ_r_* is conserved (electronic supplementary material, figure S1). $\;\bar{\;\phi }$ varied greatly because of the accumulation of inactive free ants near the rod at long time scales. However, it is freely active ants which contribute to raft dynamics. Therefore, the local packing fraction, *ϕ*, was also estimated by manually counting the number of free ants in images of regions far from the rods, with sufficient visual contrast. Free ants were distinguished from structural ants by toggling between these images and their adjacent frames to identify which ants were active.

### 2.3. Free ant trajectories

**Figure 2.**
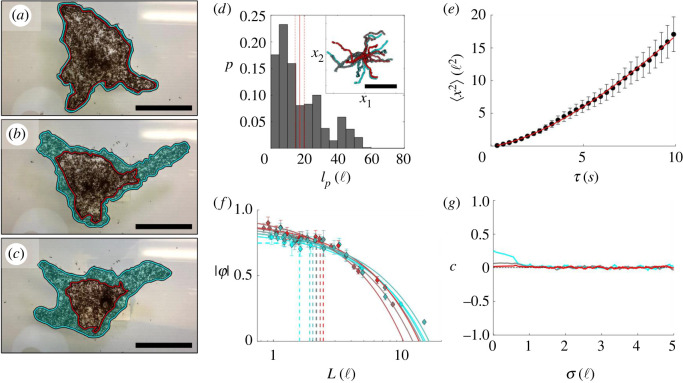
Trajectory analysis of non-edge-encountering free ants: an ant raft is depicted at the (*a*) start, (*b*) middle and (*c*) end of a 54 min duration. The red outline demarks ants that were originally in the edge of the raft at the start of the time span and the region shaded in cyan highlights the newly deposited area. Scale bars represent 20 ℓ. (*d*) The probability distribution of *l_p_* measured for 105 distinct, free ants that travelled a distance of at least 5 ℓ is displayed, with the solid and dotted vertical red lines representing the mean value and s.e., respectively (*l_p_* = 17.3 ± 2.7 ℓ). The inset displays the end-to-end trajectories of 38 free ants image-tracked over a duration of approximately 30 s to visually illustrate isotropic movement. The start of each trajectory has been centred at the origin for visual clarity and the scale bar represents 10 ℓ. (*e*) The mean ⟨*x^2^*⟩ of all samples is plotted with respect to the time interval of measurement (*τ*) for free ant trajectories tracked for at least 10 s. The red curve represents the least-squares regression fit of the form ⟨*x^2^*⟩ = *4Dτ^ξ^*. (*f*) |*φ*| is plotted with respect to the rectilinear domain size (*L*) in which it was measured for seven samples of ants over four experimental rafts. The dotted lines denote the length scales at which |*φ*|≥0.75. (*e*,*f*) Error bars represent s.e. (*g*) The moving average of *c*(*σ,τ*) is plotted with respect to the separation distance for *τ* = 0 s (cyan), *τ* = 1 s (grey) and *τ* = 10 s (red). The moving average window was set to 1 ℓ to reduce noise for transparency.

Free ants were image-tracked using ImageJ's manual image-tracking plugin. To prevent selection bias, footage was partitioned into regions of interest wherein the petiole of every free ant that entered the region was tracked frame to frame. Free ant position data, ***x****_i_*(*t*), were used to compute velocities (***v***), mean speed (*v*_0_) (electronic supplementary material, figure S2), mean square displacement (〈*x*^2^〉) (electronic supplementary material, figure S3E,F) and the local normalized order parameter (|*φ*|) (figure 2*f*) according to

2.1 $$v_{i}=[x_{i}(t+\Delta t)-x_{i}(t)]/\Delta t,$$

2.2 $$v_{0}={\left\langle \left|v_{i}\right|\right\rangle }_{N},$$

2.3 $$\left\langle x^{2}\right\rangle ={\left\langle {\left|x_{i}(t+\tau )-x_{i}(t)\right|}^{2}\right\rangle }_{N}$$

2.4 $$\mathrm{and}\;|\varphi |\;=\left|\frac{\left\langle v_{i}{\left(t\right)}_{N}\right\rangle }{{\left\langle \left|v_{i}(t)\right|\right\rangle }_{N}}\right|,$$

respectively. Here, the index *i* denotes a single ant, Δ*t* is the time between frames, *τ* is a time interval that can span multiple frames and 〈〉*_N_* denotes ensemble averaging over all *N* ants. |*φ*| was measured in successively smaller domains of square dimension *L* to determine the length scale over which order occurred (|*φ*| = 1 and |*φ*| → 0 for aligned and random motion, respectively [2]). Regions containing only one ant (where *φ* = 1 by default) were excluded. Persistence length (*l_p_*), defined as the travel distance (*l_c_*) at which correlation in an ant's trajectory is lost with itself, was also estimated according to [28]

2.5 $${\left\langle {\hat{v}}_{i,0}\cdot {\hat{v}}_{i,\tau }\right\rangle }_{N_{\tau }}=\exp\left[\frac{-l_{c}}{l_{p}}\right],$$

where ${\hat{v}}_{i,\tau }=v_{i,\tau }/|v_{i,\tau }|$ is the direction of the *i*th ant's travel at time *τ*, and ${\left\langle \;\right\rangle }_{N_{\tau }}$ denotes ensemble averaging over all *N_τ_* observations. For ideal trajectories of fixed step size and turning angle, ${\left\langle {\hat{v}}_{i,0}\cdot {\hat{v}}_{i,\tau }\right\rangle }_{N_{\tau }}$ decays exponentially with respect to *l_c_* [28], hence the form of (2.5). Although free ants do not walk ideally, a least-squares regression fit to (2.5) provides a rough estimate of *l_p_* that is useful for our purposes (see electronic supplementary material, figure S3B for extended data of ${\left\langle {\hat{v}}_{i,0}\cdot {\hat{v}}_{i,\tau }\right\rangle }_{N_{\tau }}$). Edge-encountering ants were excluded from sampling.

### 2.4. Structural contraction

**Figure 3.**
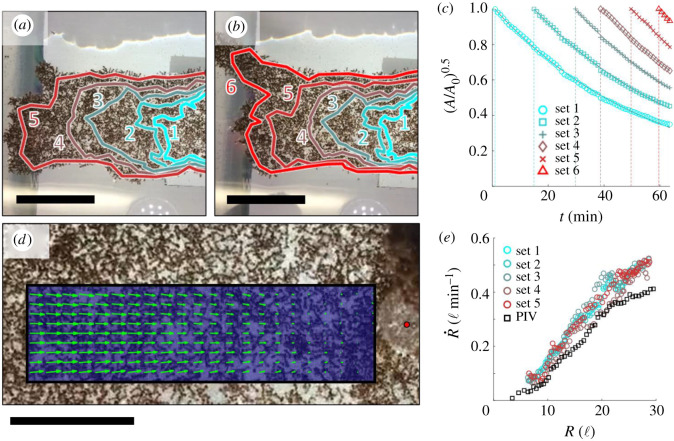
Quantifying structural retraction: a top view of an experimental raft is illustrated at the (*a*) start and (*b*) end of an approximately 8 min duration. The perimeter is traced every 15 min and outlined by numbered, coloured contours (1 represents the oldest set of ants and 6 represents the newest). The scale bars represent 20 ℓ. (*c*) The square root of the ratio *A/A_0_* is plotted with respect to time and used to estimate $\dot{\varepsilon }$ according to ${\left(A/A_{0}\right)}^{0.5}=e^{-\dot{\varepsilon }t}$. The data from each of six separately tracked sets of ants are shown, with the vertical dotted lines denoting the time at which image tracking began. $\dot{\varepsilon }$ is estimated to be 1.7–1.8% min^−1^ (*R*^2^=1.00) for all six datasets, indicating that the strain rate is approximately conserved in time. (*d*) The velocity field obtained from PIV is shown within the region of interest. To eliminate noise due to raft spin, only the radial component of the velocity (i.e. that vectored towards the anchor point of the raft denoted by a red dot) is shown. The field depicted is averaged over the full analysis duration (approx. 13 min) to reduce temporal noise. The scale bar represents 10 ℓ. (*e*) $\dot{R}$ from manual image tracking (circles in a cyan-to-red colour gradient) and PIV (black squares) is plotted with respect to *R*. Data from manual tracking represent the contractile speed of every ant sampled (i.e. the full image-tracked edge). Data from PIV are presented from every point measured in the region of interest.

We observed that the structural network contracts visibly, with structural ants appearing to flow inwards towards the stationary rod, when viewed in time-lapsed footage at 240–900× speed (electronic supplementary material, Movies S4 and S5). To quantify the contractile strain rate of the structural layer, we identified sets of structural ants originally located at the rafts' outermost edge such that the perimeter was traced with a spatial resolution of approximately 2–5 ℓ. These ants were image-tracked as they flowed inwards due to contraction (figure 3*a*,*b*). The area circumscribed by these ants (*A_r_*, outlined red in figure 2*a*–*c*; electronic supplementary material, figure S4A–C) decayed exponentially in time (*t*) according to

2.6 $$A_{r}=A_{r}^{0}e^{-2\dot{\varepsilon }t},$$

where $A_{r}^{0}$ represents the initial reference area, $\dot{\varepsilon }$ is the linear strain rate assuming isotropic contraction and the factor of 2 emerges in the exponent since the decay in area is proportionate to the one-dimensional decay-squared. $\dot{\varepsilon }$ was estimated from the coefficient of the exponential least-squares fit to (2.6). To verify isotropic contraction, $\dot{\varepsilon }$ was also estimated radially with respect to the anchoring rod as the coefficient of the linear least-squares fit to $\dot{R}(R)$ (i.e. $\dot{\varepsilon }=d\dot{R}/dR$), where *R* and $\dot{R}$ are the structural ants’ distances from the rod and speeds towards it, respectively. $\dot{R}$ was computed as $v\cdot \hat{R}$, where ***v*** was calculated via (2.1) or collected via particle image velocimetry (PIV) and $\hat{R}$ is the unit vector directed towards the anchoring rod. PIV was conducted via PIVlab [29,30] on a continuous region of interest on the largest experimental raft over a 13 min duration (figure 3*d*). Noise due to the movement of dispersed free ants on top of the structural layer was easily filtered out since free ants travel of the order of 1ℓ s^−1^, while structural contraction occurs at a rate of the order of 0.01ℓ s^−1^. Note that the free ant noise was also used to qualitatively illustrate the positions and clustering of free ants (electronic supplementary material, figure S5 and Movies S7–S10). See electronic supplementary material, figure S6 for extended $\dot{\varepsilon }$ data ($\dot{\varepsilon }>0$ represents contraction).

### 2.5. Structural exit and edge deposition rates

To quantify raft dynamics, we leveraged image-tracked data of structural ants. Given roughly conserved *ρ_r_*, the rate of structural ant exits into the free layer is

2.7 $$\delta =-2\rho_{r}\dot{\varepsilon },$$

where *δ* is measured as the number of exit events per minute per unit raft area. Again, the factor of 2 emerges because $\dot{\varepsilon }$ is the linear contraction rate, while structural exit is an areal phenomenon. Through (2.7), *δ* is measured in the bulk of the structural network. Since free ants primarily bind to the structural layer at the rafts' perimeter, this measure occurs independently of the effects of edge deposition.

We calculated the edge deposition rate per unit perimeter length (*γ*) using the newly deposited growth area (*A_g_*, shaded cyan in figure 2*a*–*c*; electronic supplementary material, figure S4A–C), taken as *A_g_* ≈ *A* − *A_r_*. Although ants may exit the structural layer in the growth zone, this was not observed to occur frequently at the perimeter among the ants that had recently transitioned, and so this estimate of *A_g_* is relatively unaffected by *δ* if the image-tracked structural ants that define *A_r_* are periodically updated (so that *A_g_* ≪ *A*). The areal growth rate was calculated via ${\dot{\;A}}_{g}=[A_{g}(t)-A_{g}(t-\Delta t)]/\Delta t$. Given constant *ρ_r_*, *γ* is

2.8 $$\gamma =\frac{{\dot{\;A}}_{g}\rho_{r}}{P},$$

where *P* is the updated raft perimeter. See electronic supplementary material, figure S4D–F for extended *α* and *δ* data, where *α* = *γP*/*A* is the edge deposition rate per unit raft area. Note that if *A* is normalized by an average area of one structural ant $\left(\rho_{r}^{-1}\right)$, then *α* and *δ* may be thought of as the areal expansion and decay rates, respectively.

### 2.6. Instabilities

Instability growth rates (*V*) and widths (*W*) were measured using ImageJ. Since the structural networks perpetually contract (including within protrusions) a pair of reference structural ants near the tip, but on opposite flanks of each protrusion, were image-tracked (electronic supplementary material, figure S7). The distance between the mean position of these reference ants and the protrusion tip (*L*) was used to calculate *V* = [*L*(*t* + Δ*t*) − *L*(*t*)]/Δ*t*. Note that local contraction was an order of magnitude smaller than tip growth $\left(L\dot{\varepsilon }\ll V\right)$, while *L* ∼ 10 ℓ. *W* was approximated via *W* ≈ *A_p_*/*L_c_*, where *A_p_* and *L_c_* are the total protrusion area and length, respectively.

## 3. Results

### 3.1. Treadmilling

The planar density of the structural network was roughly conserved throughout experiments at *ρ_r_* = 0.304 ± 0.018 ants mm^−2^, which is consistent with the value of 0.34 ± 0.02 ants mm^−2^ reported by Mlot *et al*. [11]. The mean free ant packing fraction, $\;\bar{\;\phi }$, as estimated by areal image analysis, was between 0.56 and 2.6 free ants per structural ant depending on the time of measurement and experiment. Indeed, $\;\bar{\;\phi }$ could exceed unity, indicating that ants in the free layer(s) were more numerous than those in the structural layer, consistent with the findings of Mlot *et al*. [11]. However, here this was due to local clustering of inactive ants near or on the anchoring rod. These inactive ants did not contribute to the raft dynamics reported here and even when $\;\bar{\;\phi }$ exceeded 1, raft dynamics were still observed (electronic supplementary material, figure S1C). Manual measurements confirmed that free ants in regions far from the rod remained relatively dispersed with an average density of *ρ_s_* = 0.072 ± 0.006 ants mm^−2^ and a local free ant packing fraction of *ϕ* ≈ 0.240 free ants per structural ant. This estimate of *ϕ* demonstrates that, far from the anchoring rod, free ants may exist in a dispersed state; however, even within these regions, free ant density is heterogeneous as ants form transient clusters analogous to those arising in self-propelled colloidal suspensions when *ϕ* ∼ 0.3 [31]. Regardless of density heterogeneities, we measured that free ants deposit into the edges at an average rate of *α* ≈ 0.02 deposition events min^−1^ per structural ant (or *γ* ≈ 0.29 deposition events min^−1^ per unit body length of perimeter). If the raft expanded unchecked, this would correspond to areal raft growth of the order of *α* ≈ 2% min^−1^ until the number of free ants was depleted, and a static raft area was reached. Therefore, this mechanism alone explains neither instability formation nor the dynamic treadmilling that recycles these formations over the span of hours. To better understand the full scope of what drives these features, we first examine the transport of free ants.

Previous studies modelled free ants as Brownian particles that deposit into the structural layer with constant probability upon every edge encounter, leading to isotropic raft expansion [19]. In actuality, the motion of freely active ants is not ambiently driven; rather, free ants are SPPs that actively locomote. Therefore, we characterize their trajectories in the context of active Brownian particles. First, we confirm that free ants that do not encounter the raft edges walk isotropically [11] (figure 2*d*) with *l_p_* of the order of ∼20 ℓ and *v*_0_ = 0.59 ± 0.01 ℓ s^−1^, suggesting a correlation time (*τ_r_* = *l_p_*/*v*_0_) of the order of ∼34 s [32]. Although our approximation of *l_p_* affirms that free ants can sustain self-correlated trajectories over the order of 10 ℓ, our methods of estimating *l_p_* are extrapolatory and assume that self-correlation decays exponentially. To better characterize ants’ trajectories, we also examine the mean square displacement, 〈*x*^2^〉. We find that surface ants have an average measured diffusion coefficient $\left(\bar{D}\right)$ in the range of 0.01–0.16 ℓ^2^ s^−1^ (0.1 × 10^−6^ to 1.3 × 10^−6^ m^2^ s^−1^) depending on the experiment and sample, placing the order of free ants' diffusivity near that of gaseous particles. Significantly, the ants do not diffuse randomly as previously modelled [11,19]. Instead, they diffuse anomalously according to a power law 〈*x*^2^〉 = 4*Dτ^ξ^*, where the average scaling coefficient is $\bar{\;\xi }\approx 1.48$, indicating super-diffusive behaviour (*ξ* > 1) [33] (figure 2*e*). It is worth noting that $\bar{D}$ appears to vary in both time and space for a given trajectory (hence the wide range reported here) and ants undergo interstitial periods of super- and subdiffusive behaviour (*ξ* < 1) (see electronic supplementary material, figure S3C), comparable to those which occur in the ‘run-and-tumble’ motion of swimming bacteria [34] and plankton [35] or the Lévy walks of foraging spider monkeys [36]. Such anomalous diffusion is common among motile organisms whose trajectories are influenced by both internal decisions and external stimuli. In fire ants, subdiffusive zones are likely to be caused by factors such as clustering due to volume exclusion between ants (i.e. the inability of two ants to occupy the same space) [32]. Regardless, the prevailing behaviour is that of super-diffusivity [37,38]. Super-diffusivity is not inherent to SPPs; rather, it is generally indicative of field or current-induced drift [38–40]. In this case, it is plausible that local fluxes of synchronously moving ants emerge over some length scale due to ant-to-ant (i.e. local) or ant-to-raft edge (i.e. confinement) interactions, providing early evidence that ants exhibit some degree of directed motion.

To evaluate the degree and length scale over which order in ant trajectories exists, we examine |*φ*| within successively smaller square domains of length *L* (figure 2*f*). Across seven different sets of free ants, |*φ*| scales linearly with respect to *L*^−1^. Between these samples, the length scale over which strongly ordered trajectories emerge (|*φ*| ≥ 0.75) ranges from approximately *L* ≤ 1.5 to 2.4ℓ. While some degree of synchronized motion exists within domains of the order of one to two ant body lengths, it is evident that free ants on the bulk of rafts generally movie isotropically above the length scale of a single ant. To further identify whether there exists any trajectory correlation between two neighbouring free ants (designated by indices *i* and *j*), and whether this correlation persists in time, we also examine the pairwise directional correlation between their velocities (separated by time span *τ*), according to

3.1 $$c(\sigma ,\tau )=\left\langle {\hat{v}}_{i}(t)\cdot {\hat{v}}_{j}(t+\tau )\right\rangle ,$$

where *c* → 1 indicates strong correlation (figure 2*g*), *c* ≈ 0 suggests no correlation and *c* < 0 indicates negative correlation or that the ants are walking in opposite directions. Here, ***v_i_***(*t*) and ***v_j_***(*t* + *τ*) are the velocities (***v*** = ∂***x***/∂*t*) of ant *i* (at time *t*) and ant *j* (at time *t* + *τ*), respectively, and *σ* is their ant-to-ant separation distance defined by *σ* = |***x_i_***(*t*) − ***x_j_***(*t* + *τ*)|. A delay in directional correlation is commonly used to identify leaders and followers in systems with established pairwise alignment interactions, but here *c*(*σ*, *τ*) is used to identify the length and time scales above which ants lose mutual alignment of motion [2]. Examining the spatial moving average of *c*(*σ*, *τ*) (over a window of 1ℓ to reduce noise), there appears to be no significant correlation above a length scale of ∼1ℓ, regardless of *τ*. Also, for time spans of *τ* ≥ 1 s, there appears to be no correlation in the direction of ants. Therefore, the only spatio-temporal separation for which any directional correlation occurs is *σ* < 0.75ℓ and *τ* < 1 s, suggesting that correlated movement only occurs between ants in (or nearly in) contact, and, even then, it is weak with (*c* < 0.5). The lack of correlation for *σ* > 1ℓ, despite ants' relatively long *l_p_*, suggests that ants experience no significant pairwise alignment interactions. Despite the lack of evidence for alignment interactions, we see later in this work (through measurement of *c*(*σ*, *τ*) for free ants on protrusions) how directional motion occurs in highly confined regions (wherein the dimensions of the raft are smaller than the free ants' persistence lengths), probably contributing to the runaway growth of protrusions; however, first we examine the remaining scope of mechanisms that contribute to the treadmilling dynamics which permit sustainable shape evolution. Simply reexamining figure 2*a*–*c*, it is immediately clear that the area circumscribed by the set of ants outlined in red depreciates in time, indicating that some contractile mechanism occurs within these systems.

Although ant rafts’ structural networks may appear to be amorphous solids at first glance, examination of time-lapsed footage reveals that these networks flow visibly (electronic supplementary material, Movie S4). Specifically, the structural network robustly contracts throughout the bulk at rate $\dot{\varepsilon }$. Given the fixed rod in the experimental set-up, this causes visible raft contraction towards said rod in time: $\dot{\varepsilon }\approx 1.75\text{\%}\;\min^{-1}$ (*R*^2^ = 1.00), as estimated from the areal decay rate through (2.7) (figure 3*c*). The radial contractile strain rate was calculated as $\dot{\varepsilon }=1.82\pm 0.03\text{\%}\;\min^{-1}$ from manually image-tracked data (figure 3*a*,*b*,*e*), and $\dot{\varepsilon }=1.75\pm 0.01\text{\%}\;\min^{-1}\;(R^{2}=0.97)$ from data obtained via PIV (figure 3*d,e*). The former value is within 2% of $\dot{\varepsilon }$ coarsely estimated through (2.6), while the latter value agrees with it, which suggests that the circumferential component of contraction must also be of the order of 1.8% min^−1^ and that there is no significant directional bias in contraction. Notably, measured values of $\dot{\varepsilon }$ correspond to the maximum contractile speeds of the order of just 0.01ℓ s^−1^, while free ants on top of the structural layer walk with speeds of the order of 1ℓ s^−1^. Therefore, structural contraction has a negligible effect on the previously reported trajectories of free ants.

There exists no significant correlation between $\dot{\varepsilon }$ and distance from the rod, *R* (electronic supplementary material, figure S6A–C), implying that $\dot{\varepsilon }$ is constant throughout the bulk. That contraction is both spatially constant and roughly isotropic indicates that the primary mechanism of contraction originates within the bulk structural network as opposed to entirely at a specific interface (e.g. the junction between the raft and the rod). Interestingly, despite contraction structural density, *ρ_r_* was approximately conserved throughout experiments, mandating that there exists a flux of ants out of the structural layer. Upon closer examination, we indeed observed instances of ants bound to the structural network exiting and becoming part of the free layer (electronic supplementary material, Movie S3). We quantified the exit rate, *δ*, through (2.7) to find that, across experiments, ants unparked at a rate of *δ* ≈ 2−3% min^−1^, counteracting and nearly balancing the global expansion rate *α* ≈ 2% min^−1^ measured earlier.

**Figure 4.**
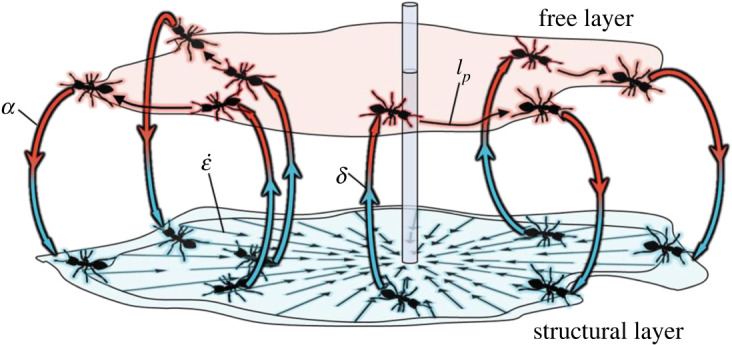
Treadmilling of fire ant rafts: contraction of the structural layer (at rate $\dot{\varepsilon }$) perpetually pulls ants in the structural network (blue) inwards, given the anchored boundary condition. Structural ants exit from the network at a rate of *δ* in the bulk and become part of the free layer of ants (red). Free ants walk directly on the top of the structural network until they encounter the perimeter of the raft. Edge-encountering ants either bank off the edge of the raft or deposit into the structural network at its perimeter at a rate of *α*. Note that free ants (denoted by the red layer) have been vertically offset from the structural ants (denoted by the blue layer) purely for visual clarity, but these two layers maintain direct contact in ant rafts.

Global raft expansion (due to the edge deposition of free ants into the structural layer) and structural contraction (concurrent with bulk dislocation of structural ants into the free layer) define the global treadmilling illustrated schematically in figure 4. This treadmilling sustains ant rafts' ability to change their shape by recycling the populations of both structural and free ants, thus also permitting the recurring formation (i.e. initiation, growth and complete retraction) of instabilities. However, the detailed causes of unstable protrusion growth remain unclear. To unveil these mechanisms, we revisit the properties of freely active ants.

### 3.2. Instabilities

**Figure 5.**
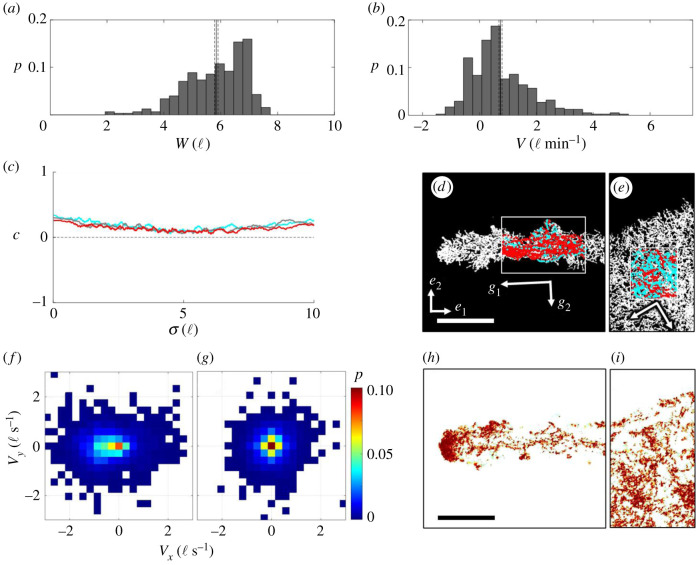
Characterizing protrusion growth and directional motion. (*a*) The distribution of 326 observations of *W*. (*b*) The distribution of 406 frame-to-frame observations of *V*. (*a*,*b*) Observations were taken over 13 distinct sample protrusions. ⟨*W*⟩ and ⟨*V*⟩ are represented by vertical lines with the dotted lines representing s.e. (*c*) The moving average of *c*(*σ,τ*) is plotted with respect to separation distance for *τ* = 0 s (cyan), *τ* = 1 s (grey) and *τ* = 10 s (red). The moving average window was set to 1 ℓ to reduce noise for transparency. (*d*,*e*) All ant trajectories within domains (*d*) on and (*e*) off a protrusion were manually image-tracked. Ants moving left and right were overlaid with red and cyan dots, respectively, to emphasize any net flux during this time span. The principal directions (eigenvectors) of *g^v^* are shown as arrows labelled *g*_1_ and *g*_2_, respectively. The magnitudes of *g*_1_ and *g*_2_ correspond to the magnitudes of the eigenvalues, 0.66 and 0.34, respectively. The sense of *g*_1_ is set to indicate the primary direction of free ant motion, and then *g*_2_ is set using right-handed sign convention. (*f*,*g*) Two-dimensional velocity probability (*p*) distributions of free ants tracked in domains (*f*) on and (*g*) off a protrusion are displayed. (*h*,*i*) Visually isolated free ants (red) (*h*) on and (*i*) off a protrusion illustrate the degree and location of clustering. All scale bars represent 10 ℓ.

Before examining the contributing factors to protrusion formation, we first quantified their characteristic growth rates and widths. Protrusions grow at an average rate of *V* = 0.74 ± 0.05 ℓ min^−1^ with an average width of 〈*W*〉 = 5.85 ± 0.06ℓ (figure 5*a*,*b*), suggesting that the areal tip growth rate per unit edge length is of the order of 〈*V*〉〈*W*〉 = 4.33 ± 0.08 ℓ^2^ min^−1^ (or 11 ants min^−1^ given that *ρ_r_* ≈ 0.3 ants mm^−2^). Normalizing this value by the approximate width of the leading tip (taken as 〈*W*〉), we see that the average tip growth rate is approximately *γ*_tip_ ≈ 1.9 min^−1^ ℓ^−1^, which is an order of magnitude higher than that of the overall raft (*γ* ≈ 0.29 min^−1^ ℓ^−1^). This disproportionate growth rate could be due to either a higher flux of free ants to the tips of protrusions or a higher probability of free ant deposition into the structural network at these locations, or both. Whether the probability of edge deposition varies by location is difficult to measure directly for two reasons. First, defining the length scale over which an ant detects the edge is not easily quantified, and so precisely recognizing edge encounters is exceedingly difficult. Second, edge accumulation effects [41] induce clustering of free ants near the edges (see figure 5*h*; electronic supplementary material, figure S5) to the extent that they become visually indistinguishable from one another in these regions and image tracking is implausible. However, characterization of free ants on the bulk of protrusions far from their tips proved feasible.

We discovered that free ants on protrusions display a high degree of directed motion, as characterized by *c*(*σ*, *τ*) from (3.1) (figure 5*c*). In fact, on protrusions, ants separated by more than 10 ℓ exhibit statistically the same directional correlation (*c* ∼ 0.1−0.2) as ants within the contact length scale. The mean value of *c*(*σ*, *τ*) across all *σ* when *τ* = 0 s is 0.170 ± 0.003, which suggests that the ants are walking on average with a nominal separation angle of approximately 80^o^. While this may seem like a large angular difference, it suggests that ants are walking on average within the same quadrant of directional orientation, indicating a net flux of ants in some direction. To confirm and identify the direction of flux, we examine a multitude of measures for free ants on a protrusion whose longitudinal axis is aligned with the first principal direction of analysis, ***e***_1_. First, we examine the metric tensor of ant velocity defined by $g^{v}={\left\langle \hat{v}⊗\hat{v}\right\rangle }_{N}$, where $\hat{v}$ represents the direction of motion of a single ant, ⊗ denotes the tensor product and the operator 〈〉*_N_* denotes averaging over the sample size, *N*. This tensor is represented by a symmetric, positive definite matrix, whose principal directions, here denoted ***g***_1_ and ***g***_2_, indicate the directions in which ant traffic is a maximum and minimum (figure 5*d*). Since ***g^v^*** is computed using unit vectors, its trace always remains unity and isotropic traffic is represented by the diagonal matrix ***g^v^*** = diag(0.5, 0.5). By contrast, anisotropic movement is represented by two distinct diagonal components of ***g^v^***. Examining ***g^v^*** in the reference coordinate axes {***e***_1_, ***e***_2_}, indeed we find that $g_{11}^{v}=0.66$ while $g_{22}^{v}=0.34$, indicating that the ants are travelling faster in the direction ***e***_1_ (which aligns with the longitudinal axis of the protrusion) than in the direction ***e***_2_. We also find that $g_{12}^{v}=g_{21}^{v}=0.01$, suggesting that the longitudinal axis is close to free ants’ principally fastest direction (with the principal components of ***g^v^*** displayed in figure 5*d* to illustrate alignment). For further visual transparency, both the 2D velocity distribution and isolated traffic of surface ants are presented in figure 5*f*,*h*, respectively. Examining the 2D velocity distribution, the maximum longitudinal component exceeds that of the transverse component. Observing figure 5*h* (see electronic supplementary material, Movie S8 for better visual clarity), the ants on the protrusion generally move longitudinally, with little transverse motion. For comparison, the metric tensor, velocity distribution and visually isolated free ant traffic far from the raft edge are depicted in figure 5*e*,*g*,*i*, respectively, and reaffirm that free ants in weak confinement move randomly. Quantitatively, isotropic movement of free ants far from the raft edge is apparent in the fact that $g_{11}^{v}\approx g_{22}^{v}$, indicating that these ants do not move preferentially in either of their principal directions.

Although ***g^v^*** indicates that free ants move primarily in line with protrusions, it reveals nothing about the sense of this movement. To identify the direction of this bias, we examine the 2D velocity distribution (figure 5*f*) and observe a slightly higher probability of ants moving towards the tip than the base at speeds up to 1 ℓ s^−1^ (i.e. the distribution is skewed slightly left). To emphasize this flux illustratively, figure 5*d* includes red and cyan points wherever an ant was recorded moving left $\left({\hat{v}}_{1}<0\right)$ or right $\left({\hat{v}}_{1}>0\right)$, respectively. Both the velocity distribution and binary plot from figure 5*d* indicate that, within the recorded time frame, more ants moved from the base to the tip of the protrusion. We hypothesize that both local tip clustering (figure 5*h*) and directional motion (figure 5*c*,*d*,*f*) of free ants on protrusions contribute to their unstable growth. To interpret experimental observations, we employ a simple model for SPPs under strong confinement that was introduced by Fily *et al.* [42]. This model prescribes that the SPPs move with some overdamped velocity and a rotational diffusion rate synonymous with *v*_0_ and $\tau_{r}^{-1}$, respectively. ‘Strong confinement’ mandates that *l_p_* of SPPs must be larger than their confining dimensions, which is satisfied by the fact that the mean persistence length of free ants is approximately three times greater than the average width of protrusions (*l_p_* ∼ 20 ℓ > 〈*W*〉 ∼ 6 ℓ) [42,43] and approximately 83% of measured ants have persistence lengths greater than 〈*W*〉 (figure 2*d*). In purely convex domains, the model predicts that SPPs glide along their confining boundaries and accumulate at regions of high local curvature [42,43].

**Figure 6.**
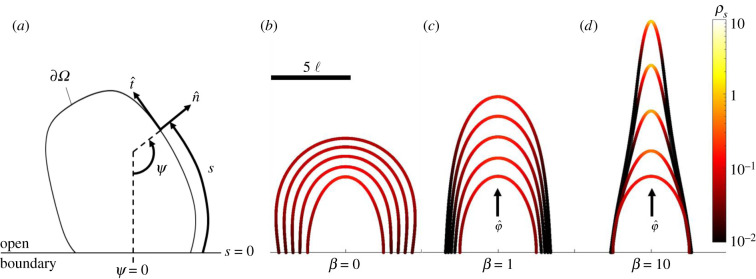
Numerical model results: (*a*) a schematic illustrates an arbitrary, convex boundary, *∂Ω*. *ψ*, *s*, $\hat{n}$ and $\hat{t}$ are all denoted at an arbitrary point along *∂Ω* to convey sign convention. (*b*–*d*) The numerical mesh is displayed from *t* = 0 s (innermost curves) to 480 s (outermost curves) in 120 s intervals for the cases of (*b*) *β* = 0, (*c*) *β* = 1 and (*d*) *β* = 10. The colour scale represents *ρ_s_* (ℓ^−1^). The scale bar represents 5 ℓ.

We implement Fily's model into a numerical framework in which half of an elliptically shaped protrusion (with a minor axis of 2.5 ℓ and major axis of 5 ℓ) is initiated. According to Fily's model, given the entirely convex domain and assuming a quasi-static state, the local density of free ants per unit edge length may be estimated by *ρ_s_* = *κ*/2*π*, where *κ* = ∂*ψ*/∂*s* is the local edge curvature, *ψ* is the orientation of the local edge normal ($\hat{n}$) and *s* denotes the curvature space along the boundary [42,43] (figure 6*a*). We impose that the local rate of directional edge deposition, ***γ***(*s*), scales linearly with the local free ant density *ρ_s_*, according to

3.2 $$\gamma =\left(a\frac{\rho_{s}}{\rho_{0}}+\gamma_{0}\right)\hat{\gamma },$$

where *a*/*ρ*_0_ defines the deposition rate's sensitivity to *ρ_s_*, *ρ*_0_ is a sensitivity parameter (in units of ℓ^−1^), *a* = *v*_0_/ℓ^2^ is a normalization constant (in units of min^−1^ ℓ^−1^), *γ*_0_ is the nominal global deposition rate (also in units of min^−1^ ℓ^−1^) and $\hat{\gamma }$ is the direction of edge deposition. Increasing *ρ*_0_ decreases the effects of *ρ_s_* on ***γ***, and increasing *γ*_0_ increases the overall edge deposition rate. We posit that $\;\hat{\;\gamma }$ has components aligned with both $\;\hat{\;n}$ and some directional bias $\left(\hat{\varphi }\right)$ such that (3.2) becomes

3.3 $$\gamma =\left[\frac{v_{0}}{\ell^{2}}\left(\frac{\rho_{s}}{\rho_{0}}\right)+\gamma_{0}\right]\;\frac{\hat{n}+\beta \;\hat{\;\varphi }}{\left|\hat{n}+\beta \;\hat{\;\varphi }\right|},$$

where ***γ*** is computed in units of deposition events per minute per unit edge length (min^−1^ ℓ^−1^). *β* is a weighting parameter that determines the extent of directional bias in edge deposition. Without bias (*β* = 0), deposition occurs normal to the edge, while high bias (*β* ≫ 0) skews this deposition in the direction of $\;\hat{\;\varphi }$. The inclusion of $\hat{\varphi }$ was initially motivated by the observed directional motion of free ants on protrusions and was set accordingly (flux of free ants occurs from base to tip such that $\hat{\varphi }=[0,1]$). *γ*_0_ was set to 0.29 min^−1^ ℓ^−1^ based on experiments. Reasonable comparison with areal experimental growth rates was coarsely achieved when *ρ*_0_ = 0.9 ℓ^−1^ (see electronic supplementary material, table S1 for a summary of parameter values). The boundary mesh was stepped in time according to *γ* = *ρ_r_*d***x***/d*t* using the forward Euler method. Note that since the observed speed of free ants (*v*_0_ ∼ 1 ℓ s^−1^) is two orders greater than that of protrusion growth (*V* ∼ 0.02 ℓ s^−1^), we posit that the steady-state assumption employed by Fily *et al.* [42] remains viable.

In the absence of biased edge deposition (figure 6*b*, *β* = 0), this model does not produce the type of protrusion growth observed for fire ants, instead predicting exaggerated outward growth with relatively diminished tip growth ($\dot{L}<0.5\;\ell \;\min^{-1}$) and edge curvature (*κ* < 0.5 ℓ^−1^). This suggests that tip clustering alone does not fully explain the runaway protrusion growth observed experimentally. We found that, as bias increases, $\dot{L}$ and *κ* also increase with reasonable comparison to experiments occurring at *β* = 1 ($\dot{L}∼1\;\ell \;\min^{-1}$ and *κ* ∼ 1 ℓ^−1^) (figure 6*c*). As bias is increased further (e.g. figure 6*d*, *β* = 10), *κ* eventually exceeds 1 ℓ^−1^, implying that the tip is less than two ants wide, which was never observed experimentally, indicating an upper limit to any biasing effects. While $\hat{\varphi }$ was initially inspired by directional motion, this model cannot specifically affirm directional motion as a first-order cause of runaway growth. Rather, it simply reveals that a bias in edge deposition, whatever its cause, significantly improves agreement.

## 4. Discussion and concluding remarks

Our experimental results reveal that dynamic shape and area changes of fire ant rafts are sustained by competing mechanisms of structural contraction and outward expansion, which together define global treadmilling. The structural network's planar density is conserved (despite contraction and any consequential areal change) owing to a flux of structural ants into the freely active surface layer. Counteracting this flux is the deposition of free ants primarily into the edge of the structural layer, driving outward expansion. That the rate of contraction slightly exceeds the rate of expansion is reflected in the eventual shrinking of overall raft areas observed after several hours owing to the accumulation of free, yet inactive ants near the anchoring rod, which slowed edge deposition. Perhaps this less active state relates to the activity cycles observed in confined, 3D aggregations of fire ants [15,16]. Ant raft evolution over longer time scales and in the context of activity may be worth examining in future work. Additionally, further consideration is warranted regarding the effects of boundary conditions on the treadmilling and instabilities observed. It remains unclear what influences, if any, the vertical rod and the height it protrudes above the water have on either of these behaviours, and a systematic sweep of this height must be conducted in order to attribute any causal relationships with raft dynamics. Furthermore, variables that may influence behaviour, such as season, the locations of ant collection and the time of day, were not considered in the scope of this study. As such, a control study may be conducted in future work to evaluate the robustness of raft dynamics under various conditions. However, here, we simply report the ant properties, treadmilling dynamics and instability characteristics as observed under the boundary conditions specified.

Similar looking finger-like instabilities (e.g. Rayleigh–Taylor [44], Kelvin–Helmholtz, Saffman–Taylor [45]) are regularly observed at fluid interfaces, owing to local property gradients (e.g. fluid density, viscosity). Although these phenomena look like fire ant protrusions, ant aggregations are distinct in several ways. First, they exist as a multi-state system whose outward expansion is driven by transport of a dispersed surface layer of free ants, as opposed to the diffusion of particles through a homogeneous bulk. Second, the dispersed layer comprises SPPs as opposed to thermally diffusing constituents. Finally, the size of individual fire ants is comparable to the size of the instabilities they form, rendering the system far from the continuum limit and introducing potential discrete size effects. Given the first two considerations, it is perhaps more appropriate to compare ant rafts with other systems of active particles in confinement. It is well demonstrated that SPPs in strong confinement accumulate in regions of local convex edge curvature [32,41,43] and sometimes phase transition into directed motion depending on the confining geometry [46]. The persistence length of ant trajectories was estimated to be of the order of 20 ℓ. This is likely to explain why free ants far from protrusions, where the confining dimensions are of the order of 20–50 ℓ, display roughly isotropic behaviour, while ants on protrusions (where *W* ∼ 6 ℓ) exhibit directional motion (figure 5*c*–*e*) and significant tip clustering (figure 5*h*). It is for this reason that we adopted the model for SPPs under strong confinement introduced by Fily *et al.* [43].

This model provides a conceptual picture of the instabilities driving protrusion initiation and growth. Citing both the model and experiments, we see that imperfections in ant rafts' edges (i.e. regions of higher edge curvature) generally host higher densities or clusters of free ants (electronic supplementary material, Movies S6–S9 and figure S5A–F) [43]. We posit that this drives an increase in the local edge deposition rate, which then strengthens the locally high curvature, introducing a positive feedback loop. Perhaps compounding this effect are factors such as the directional motion of free ants on protrusions most likely caused by the relatively small width of these features when compared with the trajectory persistence length of free ants (figure 5*c*–*e*). Observing experiments (electronic supplementary material, Movie S9 and figure S5C–F) we see that directional motion promotes additional tip clustering, thus indirectly also encouraging tip growth. While local clustering appears to accentuate growth wherever local edge symmetry is broken, it does not explain the elongated shape of some protrusions. We see from the model that if there exists a bias in the direction of edge deposition (figure 6*c*,*d*, *β* > 0), then the tip growth rates and curvatures of experimental protrusions are reasonably well replicated. One potential cause for this bias is some first-order effect from the directional motion of free ants. Indeed, where directional motion was measured experimentally (figure 5*h*), the direction of instability growth appears to be in line with said motion. Examining the model, local directional motion may also result from boundary evolution (i.e. local changes in the boundary's normal orientation, *ψ*) as growth occurs. In this case, the local orientation of SPPs, *θ*, does not necessarily align normal to the edge. According to Fily's model, this introduces a local glide speed along the boundary that depends on the difference between the ant's orientation and that of the local boundary norm according to $\dot{s}=v_{0}\cos(\theta -\psi )$. Assuming negligible change in *θ* since the persistence lengths of ants are relatively large, then this glide speed coincides with the direction of tip growth for the given boundary conditions (see electronic supplementary material, figure S8) and provides one possible explanation for the bias in directional tip growth observed. Besides bulk directional motion seen in experiments and local directional gliding predicted by the model, we also observed cases of protrusion initiation in regions of low boundary curvature that seemingly occurred when many ants approach the local edge simultaneously (electronic supplementary material, Movie S10 and figure S5G,H). In these cases, spontaneous directional motion appears to antecede locally strong confinement and wall accumulation, suggesting that directional motion may sometimes be the original cause of asymmetric growth. Similar systems of SPPs in confinement display tether-like growths attributed to local cooperative effects. For example, Vutukuri *et al.* [47] revealed that Janus particles inside 3D lipid vesicles initiate protrusions when multiple Janus particles undergo spontaneous synchronous motion and apply a cooperative local force on the vesicle wall [47]. Remarkably, these growths emerge in the absence of centralization or external gradients. Whether this is also the case in fire ants remains to be seen, as other potential causes of biased edge deposition, such as environmental cues or local pheromone signals (analogous to chemotactic agents [5]), have not been ruled out.

While the model employed here helps interpret possible first- and second-order effects driving instabilities, it still possesses limitations in the context of fire ants. For example, it assumes a dilute system without local interactions. This presupposes that jamming does not occur and SPPs in strong confinement congregate entirely at the boundaries. Furthermore, this model assumes that density gradients reach a quasi-static state. However, this is not the case for fire ants, which exist at packing fractions between approximately 0.2 and 0.8 free ants per structural ant, with local concentrations evolving ceaselessly. At these concentrations, ants appear to cluster and jam at certain locations, reflecting the phases that evolve in systems of SPPs with volume exclusion interactions [32]. Furthermore, this model steps the boundary continuously to preserve smooth functions in curvature space (*ψ*, *κ* and ∂*κ*/∂*s*) despite the acknowledged potential for discrete size effects in real ants. Finally, it coarse-grains directional deposition bias through $\hat{\varphi }$ and cannot elucidate its underlying cause(s). This motivates future work in which we will employ discrete, agent-based modelling to better understand the physics of this biological system, while also providing swarm roboticists and engineers with distilled, ant-inspired rules that may achieve complex functional tasks.

## Data accessibility

The data supporting the findings of this publication and codes used to generate figures and statistics are publicly available at https://doi.org/10.25810/bh4h-k637 [48].
